# Supplementary material for: COUP-TFII-mediated reprogramming of the vascular endothelium counteracts tumor immune evasion
Source: Nat Commun. 2025 Aug 12;16:7457. doi: 10.1038/s41467-025-62399-1 (PMC12343902; doi:10.1038/s41467-025-62399-1)
Supplement: Supplementary file 1 — Supplementary Information [file 41467_2025_62399_MOESM1_ESM.pdf]

Figure S1

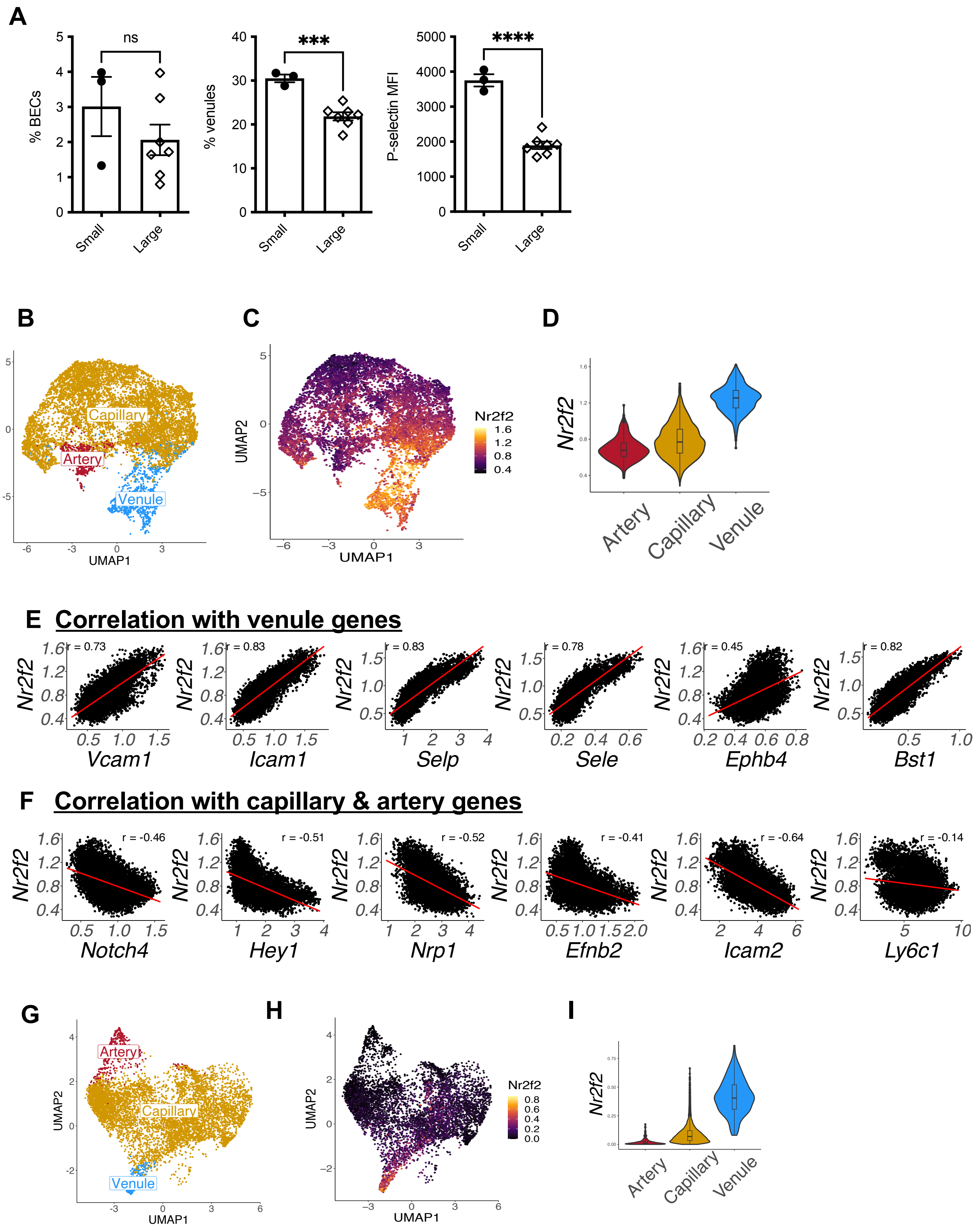
A
% BECs
ns
% venules
***
P-selectin MFI
****
Small
Large
B
Capillary
Artery
Venule
UMAP1
UMAP2
C
Nr2f2
D
Artery
Capillary
Venule
E Correlation with venule genes
r = 0.73
r = 0.83
r = 0.83
r = 0.78
r = 0.45
r = 0.82
Vcam1
Icam1
Selp
Sele
Ephb4
Bst1
F Correlation with capillary & artery genes
r = -0.46
r = -0.51
r = -0.52
r = -0.41
r = -0.64
r = -0.14
Notch4
Hey1
Nrp1
Efnb2
Icam2
Ly6c1
G
H
I

Supplementary Figure 1. Tumor expansion downregulates COUP-TFII-expressing venular endothelial cells.

(A) Flow cytometric quantification of the abundance of BECs, the representation of P-selectin+ venules among BECs, and the BEC expression fluorescence intensity of P-selectin in orthotopic KPC tumors. (n=3-7/group).

(B-C) scRNAseq UMAP plots of BECs from orthotopic PyMT-B6 tumors colored by EC subsets and Nr2f2 (COUP-TFII) expression.

(D) Violin plot of Nr2f2 expression in BEC subsets from orthotopic PyMT tumors.

(E-F) scRNAseq dot plots showing correlation between Nr2f2 expression and capillary and venule markers from orthotopic PyMT tumors.

(G-H) scRNAseq UMAP plots of BECs from orthotopic KPC tumors colored by EC subsets or Nr2f2 expression.

(I) Violin plot of Nr2f2 expression in BEC subsets from orthotopic KPC tumors in (b).

* $p<0.05$, ** $p<0.01$, *** $p<0.001$, ****$p<0.0001$ by unpaired Student's t-test. Error bars indicate s.e.m.

Figure S2

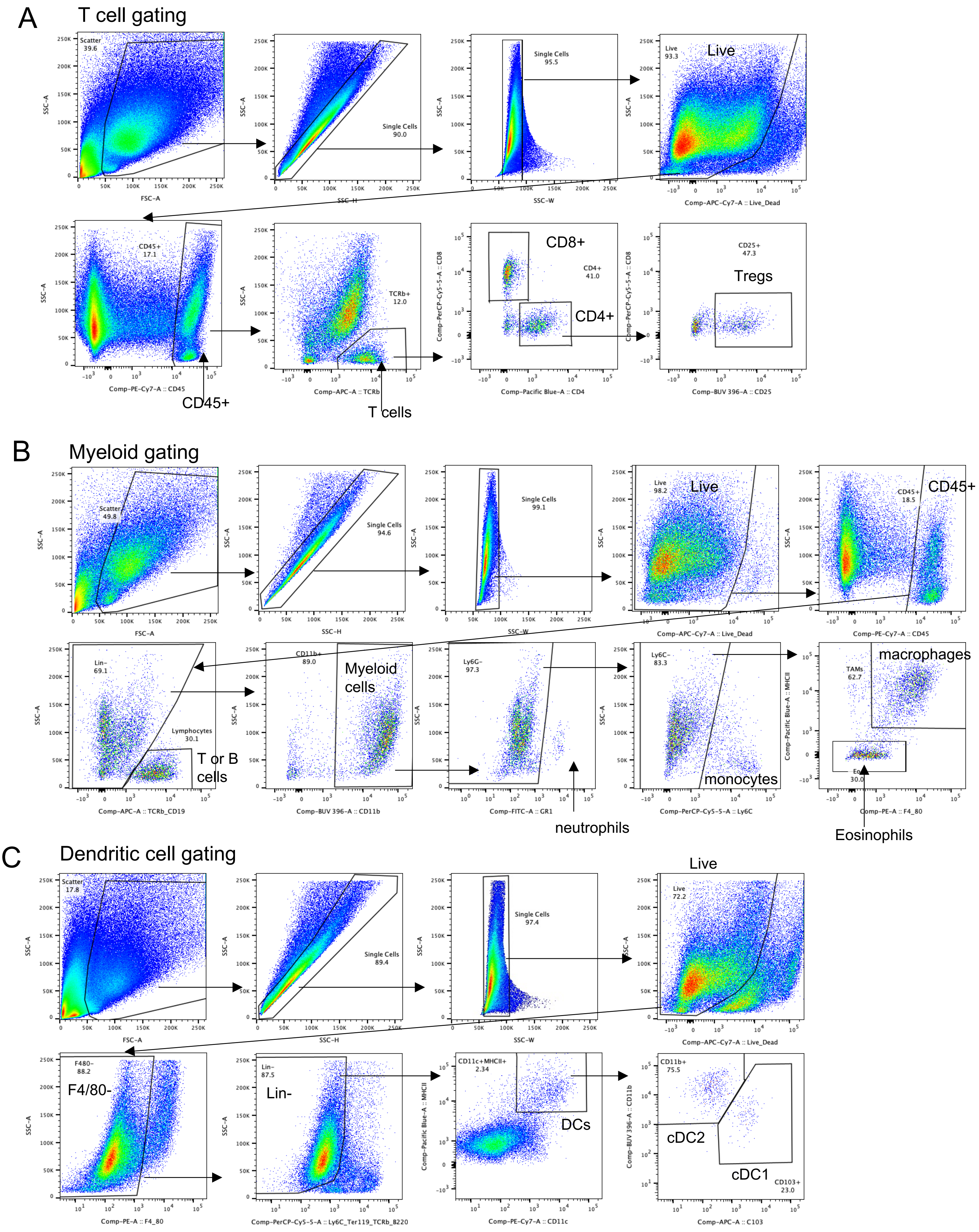
A
T cell gating
Scatter
39.6
Single Cells
90.0
Single Cells
95.5
Live
93.3
Live
CD45+
17.1
CD45+
TCRb+
12.0
T cells
CD8+
CD4+
41.0
CD4+
CD25+
47.3
Tregs
FSC-A
SSC-A
SSC-H
SSC-W
Comp-APC-Cy7-A :: Live_Dead
Comp-PE-Cy7-A :: CD45
Comp-APC-A :: TCRb
Comp-Pacific Blue-A :: CD4
Comp-PerCP-Cy5-5-A :: CD8
Comp-BUV 396-A :: CD25
B
Myeloid gating
Scatter
49.8
Single Cells
94.6
Single Cells
99.1
Live
98.2
Live
CD45+
18.5
CD45+
Lin-
69.1
Lymphocytes
30.1
T or B cells
CD11b+
89.0
Myeloid cells
Ly6G-
97.3
neutrophils
Ly6C-
83.3
monocytes
TAMs
62.7
macrophages
Eo
30.0
Eosinophils
Comp-APC-A :: TCRb_CD19
Comp-BUV 396-A :: CD11b
Comp-FITC-A :: GR1
Comp-PerCP-Cy5-5-A :: Ly6C
Comp-PE-A :: F4_80
Comp-Pacific Blue-A :: MHCII
C
Dendritic cell gating
Scatter
17.8
Single Cells
89.4
Single Cells
97.4
Live
72.2
Live
F480-
88.2
F4/80-
Lin-
87.5
Lin-
CD11c+MHCII+
2.34
DCs
CD11b+
75.5
cDC2
cDC1
CD103+
23.0
Comp-PE-A :: F4_80
Comp-PerCP-Cy5-5-A :: Ly6C_Ter119_TCRb_B220
Comp-PE-Cy7-A :: CD11c
Comp-Pacific Blue-A :: MHCII
Comp-APC-A :: C103
Comp-BUV 396-A :: CD11b

Supplementary Figure 2. Gating strategies for tumor-infiltrating immune cells.

(a) Gating strategies for T cells (CD45+ TCRb+ and CD4 or CD8+).

(b) Gating strategy for myeloid cells (CD45+ TCRb- CD19- CD11b+), including neutrophils (GR1^{high}), monocytes (GR1$^{low/neg}$, Ly6C^{high}), TAMs (GR1- Ly6C$^{low/neg}$, F4/80+ MHCII+), and eosinophils (GR1- Ly6C$^{low/neg}$ F4/80+ MHCII- SiglecF+).

(c) Gating strategy for dendritic cells (lineage (Ly6C/B220/TCRb-Ter119)- F4/80- CD11c+ MHCII+, including cDC1 (CD103+) and cDC2 (CD11b+)).

Figure S3

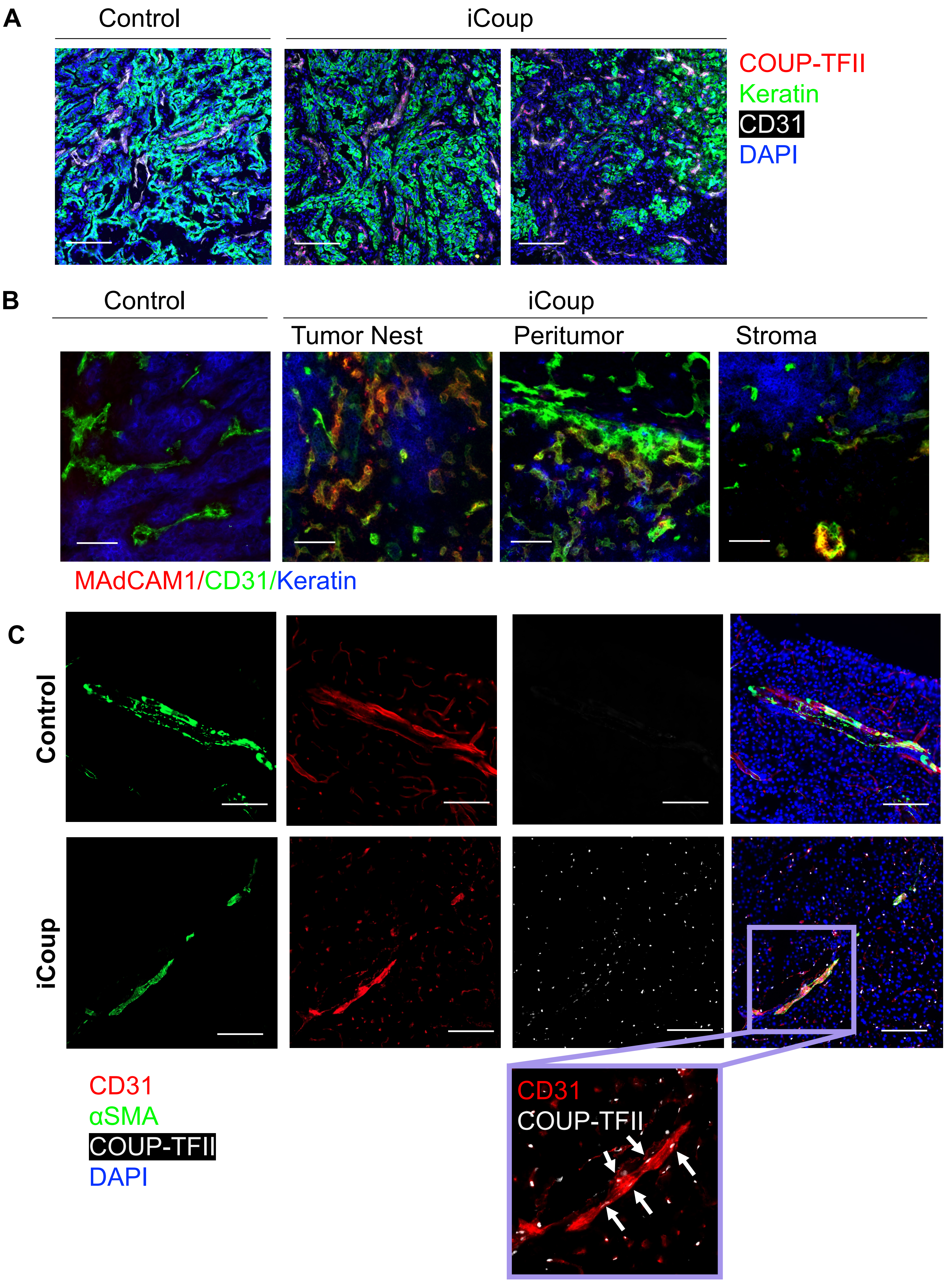
A
Control
iCoup
COUP-TFII
Keratin
CD31
DAPI
B
Control
iCoup
Tumor Nest
Peritumor
Stroma
MAdCAM1/CD31/Keratin
C
Control
iCoup
CD31
COUP-TFII
CD31
αSMA
COUP-TFII
DAPI

Supplementary Figure 3. Characterization of blood vessels with ectopic COUP-TFII expression.

(A) Representative immunofluorescence images of COUP-TFII-expressing endothelial cells (CD31+) and tumor cells (keratin+) in the intratumor and peritumor/stroma regions of orthotopic KPC tumors established in iCoup mice. Scale bars represent 50 μm.

(B) Representative immunofluorescence images of *de novo* reprogrammed ECs (marked by MAdCAM1+) in the tumor nest, peritumor, and stromal regions of orthotopic KPC tumors established in iCoup mice. Scale bars represent 20 μm.

(C) Representative immunofluorescence images of COUP-TFII-expressing arterial endothelial cells covered by smooth muscle cells (αSMA) in the pancreas adjacent to orthotopic KPC tumors. Arrows indicate arterial ECs ectopically expressing COUP-TFII. Scale bars represent 50 μm.

Figure S4

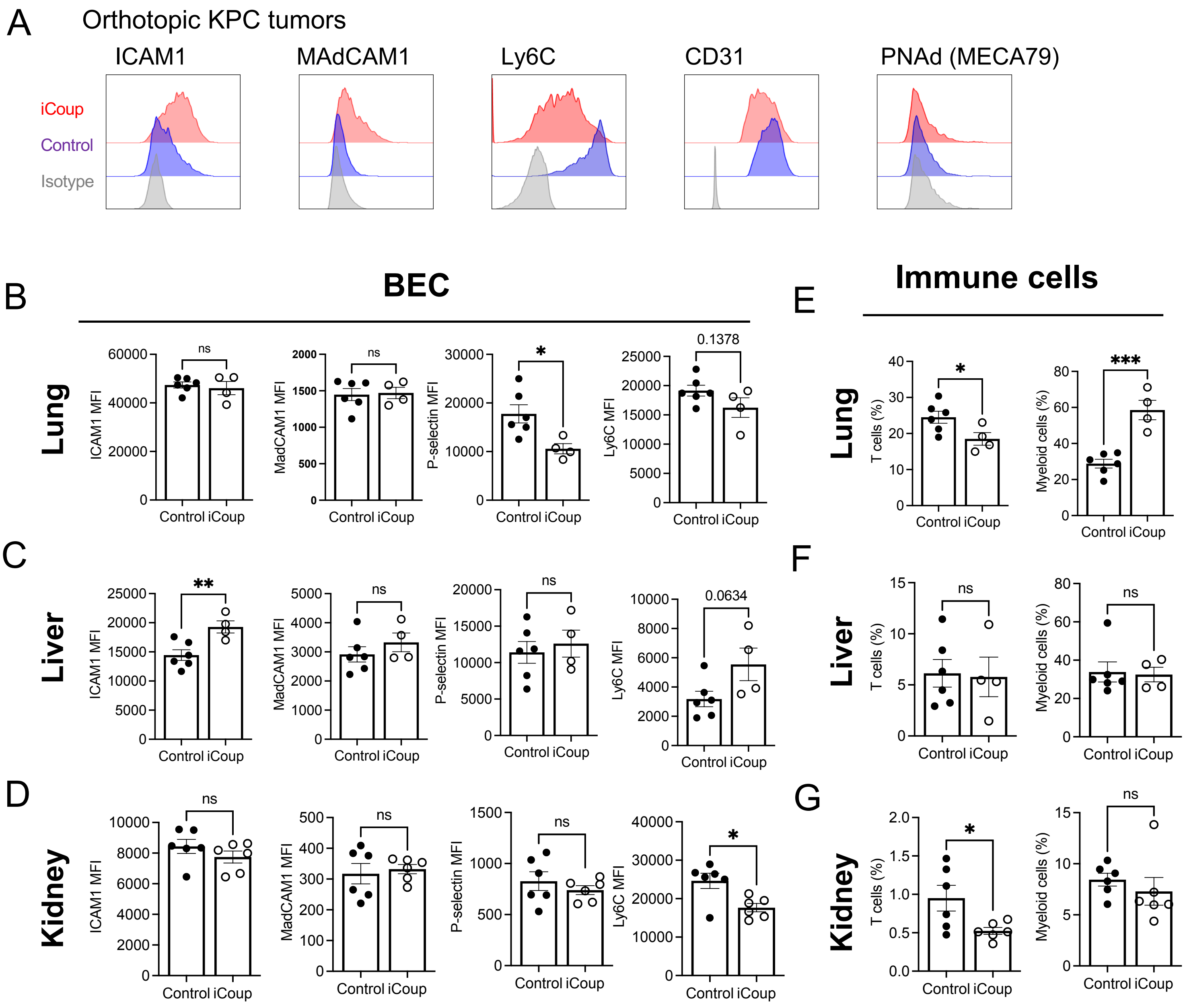
A
Orthotopic KPC tumors
ICAM1
MAdCAM1
Ly6C
CD31
PNAd (MECA79)
iCoup
Control
Isotype
B
BEC
Lung
ICAM1 MFI
MadCAM1 MFI
P-selectin MFI
Ly6C MFI
ns
*
0.1378
Control iCoup
C
Liver
**
0.0634
D
Kidney
E
Immune cells
T cells (%)
Myeloid cells (%)
***
F
G

Supplementary Figure 4. Ectopic COUP-TFII in TECs drives capillary-to-venule reprogramming but effects on other vascular beds are varied.

(A) Representative histograms of indicated markers in BECs from orthotopic KPC tumors established in control and iCoup mice. Representative of more than 5 independent experiments.

(B-D) Flow cytometry quantification of indicated markers in BECs from normal lung, liver, and kidney in control and iCoup mice. (n=4-6/group).

(E-G) Flow cytometry quantification of indicated immune cells in normal lung, liver, and kidney in control and iCoup mice. (n=4-6/group).

Figure S5

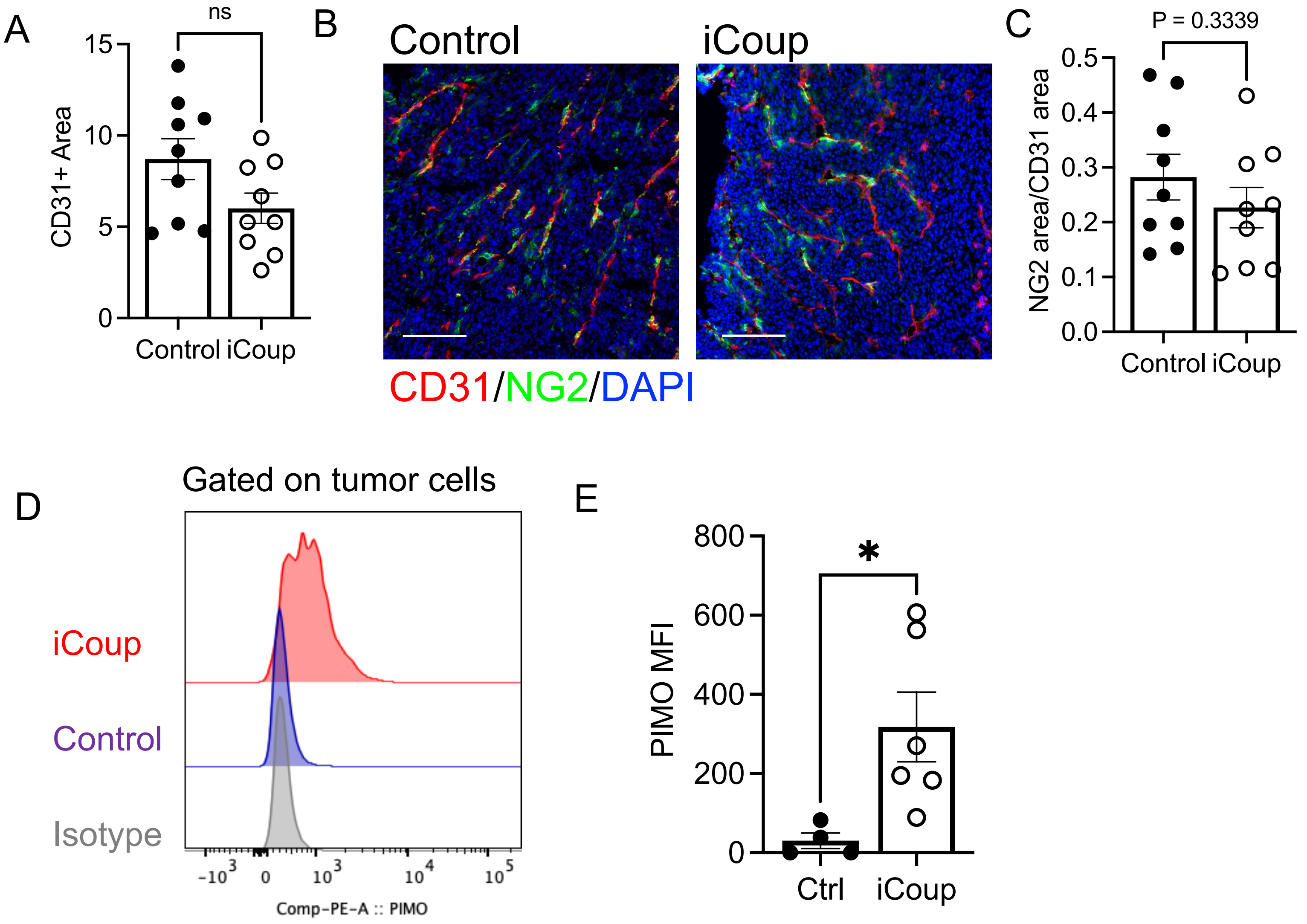
A
ns
CD31+ Area
15
10
5
0
Control
iCoup
B
Control
iCoup
CD31/NG2/DAPI
C
P = 0.3339
NG2 area/CD31 area
0.5
0.4
0.3
0.2
0.1
0.0
Control
iCoup
D
Gated on tumor cells
iCoup
Control
Isotype
Comp-PE-A :: PIMO
E
*
PIMO MFI
800
600
400
200
0
Ctrl
iCoup

Supplementary Figure 5. Ectopic COUP-TFII in TECs upregulates tumor hypoxia but not pericyte recruitment.

(A) Immunofluorescence quantification of CD31+ areas in orthotopic PyMT tumors established in control and iCoup mice. (n=9 animals/group).

(B) Representative immunofluorescence images of endothelial cells (CD31+) and pericytes (NG2+) in orthotopic PyMT tumors established in control and iCoup mice. Scale bars represent 50 μm.

(C) Ratio of NG2+ area over CD31+ area in **(B)**.

(D-E) Representative histograms of pimonidazole staining in tumor cells (CD45-CD31-podoplanin-EpCAM+) from orthotopic KPC tumors established in control and iCoup mice. Representative of 3 independent experiments. (n=4-6/group).

Figure S6

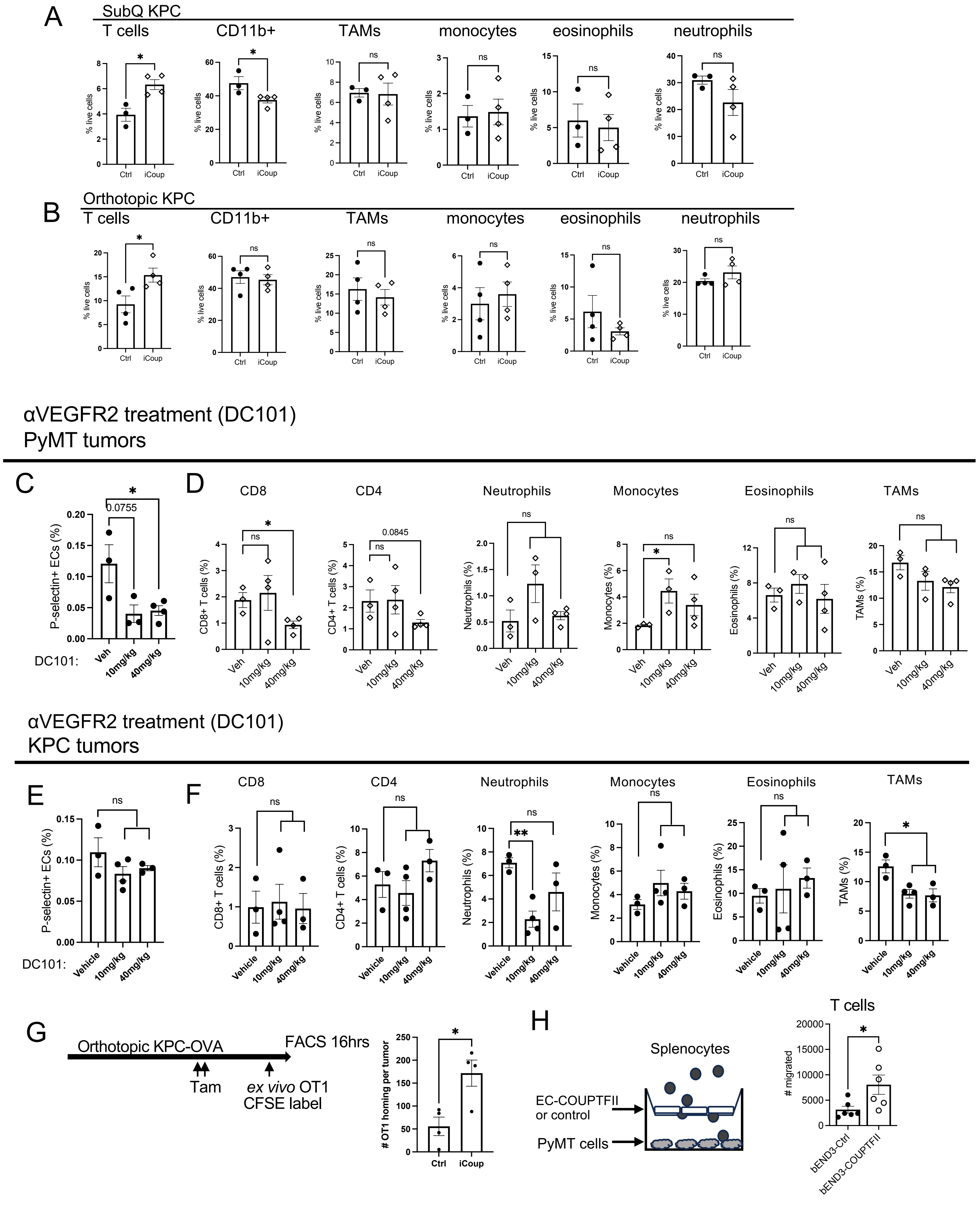
A
SubQ KPC
T cells
CD11b+
TAMs
monocytes
eosinophils
neutrophils
% live cells
Ctrl
iCoup
ns
*
B
Orthotopic KPC
T cells
CD11b+
TAMs
monocytes
eosinophils
neutrophils
αVEGFR2 treatment (DC101)
PyMT tumors
C
P-selectin+ ECs (%)
0.0755
DC101:
Veh
10mg/kg
40mg/kg
D
CD8
CD4
Neutrophils
Monocytes
Eosinophils
TAMs
CD8+ T cells (%)
CD4+ T cells (%)
0.0845
Neutrophils (%)
Monocytes (%)
Eosinophils (%)
TAMs (%)
αVEGFR2 treatment (DC101)
KPC tumors
E
F
Vehicle
**
G
Orthotopic KPC-OVA
FACS 16hrs
Tam
ex vivo OT1
CFSE label
OT1 homing per tumor
Ctrl
iCoup
H
Splenocytes
EC-COUPTFII
or control
PyMT cells
T cells
migrated
bEND3-Ctrl
bEND3-COUPTFII

Supplementary Figure 6. Ectopic COUP-TFII in TECs upregulates recruitment, while VEGFR blockade did not increase T cell infiltration in KPC tumors.

(A-B) Flow cytometry quantification of tumor-infiltrating leukocytes in subcutaneous (A) and orthotopic (B) KPC tumors in iCoup and control mice. (n=3-4/group). Representative of 3 independent experiments.

(C-F) Flow cytometric quantification of indicated populations in orthotopic PyMT **(C-D)** and KPC **(E-F)** tumors after treatment with indicated doses of VEGFR2 antibody (clone DC101). (n=3/group).Representative of 2 independent experiments.

(G) Schematics and flow cytometric quantification of OT1 T cell homing to orthotopic KPC-OVA tumors. (n=4/group). Representative of 2 independent experiments.

(H) Schematic and flow cytometric quantification of splenic T cell trans-endothelial migration to tumor cells across monolayers of bEND3 cells overexpressing COUP-TFII or vector control. (n=6/group). Representative of 5 independent experiments.

* $p< 0.05$ ** $p<0.01$ by unpaired Student's t-test. ns denotes not significant. Error bars indicate s.e.m.

Supplementary Table 1. Sequences of QPCR primers

Targets	Forward Primer	Reverse Primer
Gapdh	TGACCTCAACTACATGGTCTACA	CTTCCCATTCTCGGCCTTG
Hprt	TCAGTCAACGGGGGACATAAA	GGGGCTGTACTGCTTAACCAG
Nr2f2	TCAACTGCCACTCGTACCTG	CCATGATGTTGTTAGGCTGCAT
Ephb4	GAGAGCGAAGCTGATACGGC	CTCGGCAGCGTACAGCATAA
Sele	GACCTGGAACCCTACATGGAT	TTATGCAAACACTTCTCGGCT
Madcam1	CCTGGCCCTAGTACCCTACC	CCGTACAGAGAGGATACTGCTG
Rbpj	CTCCACCCAAACGACTCACTA	TCCAACCACTGCCCATAAGATA
Podxl	AGGTGGTCAACCTTAATGGGG	TCATCCTTGGTCAAGTTGTCCA
Cdh5	CACTGCTTTGGGAGCCTTC	GGGGCAGCGATTCATTTTTCT
Eng	CCCTCTGCCCATTACCCTG	GTAAACGTCACCTCACCCCTT
Ccl2	ATTCTGTGACCATCCCCTCAT	TGTATGTGCCTCTGAACCCAC
Ccl5	GCTGCTTTGCCTACCTCTCC	TCGAGTGACAAACACGACTGC
Cxcl10	CCAAGTGCTGCCGTCATTTTC	GGCTCGCAGGGATGATTTCAA
